# Precise Species Identification for Acinetobacter: a Genome-Based Study with Description of Two Novel Acinetobacter Species

**DOI:** 10.1128/mSystems.00237-21

**Published:** 2021-05-26

**Authors:** Jiayuan Qin, Yu Feng, Xiaoju Lü, Zhiyong Zong

**Affiliations:** a Center of Infectious Diseases, West China Hospital, Sichuan University, Chengdu, Sichuan, China; b Division of Infectious Diseases, State Key Laboratory of Biotherapy, Chengdu, Sichuan, China; c Center for Pathogen Research, West China Hospital, Sichuan University, Chengdu, Sichuan, China; d Department of Infection Control, West China Hospital, Sichuan University, Chengdu, Sichuan, China; University of Delhi

**Keywords:** Acinetobacter, genome analysis, phylogenetic analysis, quasispecies, species

## Abstract

The genus Acinetobacter comprises species with ecological significance and opportunistic pathogens and has a complicated taxonomy. Precise species identification is a foundation for understanding bacteria. In this study, we found and characterized two novel Acinetobacter species, namely, Acinetobacter tianfuensis sp. nov. and Acinetobacter rongchengensis sp. nov., based on phenotype examinations and genome analyses of the two strains WCHAc060012^T^ and WCHAc060115^T^. The two strains had ≤89.69% (mean, 79.28% or 79.72%) average nucleotide identity (ANI) and ≤36.4% (mean, 20.89% or 22.19%) *in silico* DNA-DNA hybridization (*is*DDH) values compared with each other and all known Acinetobacter species. Both species can be differentiated from all hitherto known Acinetobacter species by a combination of phenotypic characteristics. We found that Acinetobacter pullorum B301^T^ and Acinetobacter portensis AC 877^T^ are actually the same species with 98.59% ANI and 90.4% *is*DDH values. We then applied the updated taxonomy to curate 3,956 Acinetobacter genomes in GenBank and found that 6% of Acinetobacter genomes (*n* = 234) are required to be corrected or updated. We identified 56 novel tentative Acinetobacter species, extending the number of Acinetobacter species to 144, including 68 with species names and 76 unnamed taxa. We also found that ANI and the average amino acid identity (AAI) values among type or reference strains of all Acinetobacter species and taxa are ≥76.97% and ≥66.5%, respectively, which are higher than the proposed cutoffs to define the genus boundary. This study highlights the complex taxonomy of Acinetobacter as a single genus and the paramount importance of precise species identification. The newly identified unnamed taxa warrant further studies.

**IMPORTANCE** Acinetobacter species are widely distributed in nature and are of important ecological significance and clinical relevance. In this study, first, we significantly update the taxonomy of Acinetobacter by reporting two novel Acinetobacter species, namely, Acinetobacter tianfuensis and Acinetobacter rongchengensis, and by identifying Acinetobacter portensis as a synonym of Acinetobacter pullorum. Second, we curated Acinetobacter genome sequences deposited in GenBank (*n* = 3,956) using the updated taxonomy by correcting species assignations for 6% (*n* = 234) genomes and by assigning 94 (2.4%) to 56 previously unknown tentative species (taxa). Therefore, after curation, we further update the genus Acinetobacter to comprise 144 species, including 68 with species names and 76 unnamed taxa. Third, we addressed the question of whether such a large number of species should be divided in different genera and found that Acinetobacter is indeed a single genus. Our study significantly advanced the taxonomy of Acinetobacter, an important genus with science and health implications.

## INTRODUCTION

The genus Acinetobacter, first proposed by Brisou and Prévot (1), is a highly diverse group. Members of the genus Acinetobacter are distributed widely in soil and water (2) and possess versatile metabolic capabilities for the degradation of various compounds, such as long-chain dicarboxylic acids and aromatics, and actively participate in the nutrient cycle in the ecosystem (3, 4). Some Acinetobacter species are also well-known opportunistic pathogens causing a variety of human infections (5–8). Precise species assignation lays a foundation for understanding the habitat, epidemiology, pathogenesis, and microbiological features of bacteria and has important implications for health, industry, and science, while updated and curated taxonomic assignment is the premise of precise species identification (9, 10). Before the present study, the genus Acinetobacter included 67 species with validly published names (11) and 20 additional Acinetobacter species with tentative species designations (www.szu.cz/anemec/Classification.pdf). Validly published names refer to those published in the International Journal of Systematic and Evolutionary Microbiology, the official journal of the International Committee on Systematics of Prokaryotes, including its validation lists (12). New Acinetobacter species are continuingly being reported, and the number of Acinetobacter species increases every year, with 6 novel species in 2017, 3 in 2018, 4 in 2019. and 9 in 2020 (11, 13–18). However, the taxonomy of Acinetobacter is complicated by the presence of synonyms (19–22). In addition, it is not uncommon that bacterial genomes deposited in GenBank are mislabeled for species assignations (10, 23, 24) (https://help.ezbiocloud.net/type-strain-and-reference-strain/). Therefore, there is a need to update the taxonomy of Acinetobacter and to curate the species assignations of Acinetobacter genome sequences deposited in GenBank.

Here, we report two novel Acinetobacter species, namely, Acinetobacter tianfuensis sp. nov. and Acinetobacter rongchengensis sp. nov., based on phenotypic characterization and genomic analysis. We updated the Acinetobacter taxonomy and found a pair of synonyms, Acinetobacter pullorum and Acinetobacter portensis, which has not been identified before. We then used the updated taxonomy to curate 5,997 Acinetobacter genomes available in GenBank (accessed by 1 August 2020), and we identified 56 previously unknown tentative species designations.

## RESULTS

### Identification of two novel Acinetobacter species, namely, Acinetobacter tianfuensis and Acinetobacter rongchengensis.

Two Acinetobacter strains, namely, WCHAc060012^T^ and WCHAc060115^T^, were recovered from hospital sewage using an Acinetobacter chromogenic agar plate in 2018. We obtained the nearly complete 16S rRNA gene sequences (1,352 bp) of the two strains using PCR with the universal primers 27F and 1492R (25) and Sanger sequencing as described previously (26) for preliminary species identification. Comparison of the 16S rRNA gene sequences in the EzBioCloud database (27) and the 16S rRNA gene sequence-based phylogenetic tree (see Fig. S1 in the supplemental material) revealed that the two strains indeed belonged to the genus Acinetobacter. Strains WCHAc060012^T^ and WCHAc060115^T^ had the highest identity of 16S rRNA gene sequences with Acinetobacter chengduensis WCHAc060005^T^ (98.96%; accession no. MK796535) and Acinetobacter chinensis WCHAc010005^T^ (98.05%; accession no. NR_165666), respectively. However, it is well known that analysis based on the 16S rRNA sequence is insufficient for accurate taxonomic assignment (28). We then compared partial *rpoB* sequences (861 bp) of the two strains with those of Acinetobacter type strains. The two strains were also distinct from all known Acinetobacter species and formed two evolutionary clades in the phylogenetic tree based on partial *rpoB* gene sequences (see Fig. S2 in the supplemental material). Strain WCHAc060012^T^ had the highest identity of the partial *rpoB* sequence with Acinetobacter wanghuae dk386^T^ (89.08%), while WCHAc060115^T^ had the highest identity with Acinetobacter piscicola KCTC 62134^T^ (95.23%).

10.1128/mSystems.00237-21.1 FIG S1 Maximum likelihood phylogenetic tree based on 16S rRNA gene sequences (1,352 bp) of WCHAc060012^T^, WCHAc060115^T^, and type strains of Acinetobacter species with validly published names. The sequence of Moraxella lacunata ATCC 17967^T^ (GenBank accession no. AF005160) was used as the outgroup. Bootstrap values (≥50%) after 1,000 resamplings are indicated at branch nodes. Shown in parentheses are the DDBJ/ENA/GenBank accession no. for 16S rRNA gene sequences or whole-genome sequences. Bar, 0.2 substitutions per nucleotide position. Download FIG S1, PDF file, 2.6 MB. Copyright © 2021 Qin et al. 2021 Qin et al. https://creativecommons.org/licenses/by/4.0/ This content is distributed under the terms of the Creative Commons Attribution 4.0 International license.

10.1128/mSystems.00237-21.2 FIG S2 Maximum likelihood phylogenetic tree based on partial *rpoB* (861 bp) gene sequences of WCHAc060012^T^, WCHAc060115^T^, and type strains of Acinetobacter species with validly published names. Evolutionary distances were computed using Kimura’s two-parameter model. Moraxella lacunata ATCC 17967^T^ was used as the outgroup. Bootstrap values ≥ 50% based on 1,000 resamplings are shown. Shown in parentheses are the DDBJ/ENA/GenBank accession no. for *rpoB* gene sequences or whole-genome sequences. Bar, 0.2 substitutions per nucleotide position. Download FIG S2, PDF file, 2.3 MB. Copyright © 2021 Qin et al. 2021 Qin et al. https://creativecommons.org/licenses/by/4.0/ This content is distributed under the terms of the Creative Commons Attribution 4.0 International license.

**FIG 1:**
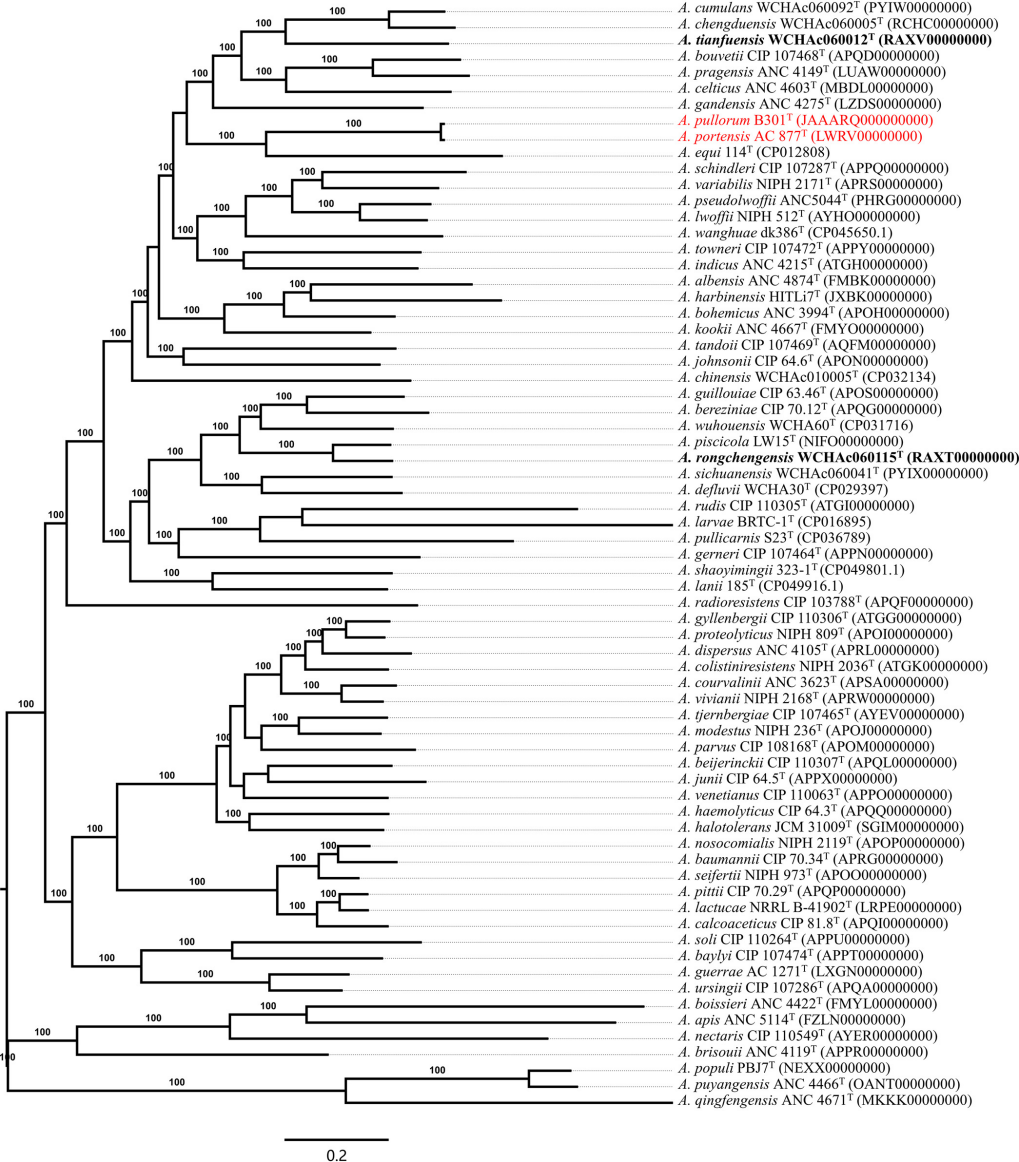
Phylogenomic tree of WCHAc060012^T^, WCHAc060115^T^, and type strains of Acinetobacter species with validly published names. The phylogenomic tree was inferred based on the alignment of 1,397 core genes. WCHAc060012^T^ and WCHAc060115^T^ are highlighted in bold. *A. pullorum* and *A. portensis*, a pair of synonyms, are highlighted in red. DDBJ/ENA/GenBank accession no. are shown in parentheses and 100% bootstraps are indicated. Bar, 0.2 changes per nucleotide position.

To further explore their precise species assignations, the two strains were subjected to whole-genome sequencing using Illumina HiSeq X10 platform. For strain WCHAc060012^T^, 6,401,206 reads and 1.92 giga-bases (Gb) were generated with an actual 549.9× coverage, which were assembled into a 3.5-Mb draft genome sequence containing 116 contigs (*N*_50_, 77,978 bp) with a G+C content of 42.3% . For strain WCHAc060115^T^, 5,479,547 reads and 1.64 Gb were generated with an actual 396.8 × coverage, which were assembled into a 4.1-Mb draft genome sequence containing 248 contigs (*N*_50_, 68,539 bp) with a G+C content of 37.7%. We determined the average nucleotide identity (ANI) values between WCHAc060012^T^ and WCHAc060115^T^ and between the two strains and type strains of all Acinetobacter species. Compared with type strains of all Acinetobacter species, ANI values of WCHAc060012^T^ ranged from 77.09% (Acinetobacter puyangensis ANC 4466^T^) to 82.70% (Acinetobacter cumulans WCHAc060092^T^), while those of WCHAc060115^T^ ranged from 77.71% (*A. puyangensis* ANC 4466^T^) to 89.69% (Acinetobacter *piscicola* LW15^T^) (Table 1). The ANI value between WCHAc060012^T^ and WCHAc060115^T^ was 79.46% (Table 1). These ANI values are well below the 95% to 96% threshold used to define bacterial species (29). We then performed *in silico* DNA-DNA hybridization (*is*DDH) analyses for WCHAc060012^T^, WCHAc060115^T^, and type strains of all Acinetobacter species. The *is*DDH values of WCHAc060012^T^ and type strains of all Acinetobacter species were 19.2% to 23.4%, while those of WCHAc060115^T^ and type strains of all Acinetobacter species were 20.0% to 36.4% (Table 1), which are far below the 70% cutoff used to define a species (30, 31). The *is*DDH value between WCHAc060012^T^ and WCHAc060115^T^ was 21.7% (Table 1). Both ANI and *is*DDH analyses clearly indicate that the two strains represent two novel Acinetobacter species. In the phylogenomic tree based on core genes (Fig. 1), WCHAc060012^T^ and WCHAc060115^T^ are most closely related to *A. cumulans* WCHAc060092^T^ and *A. piscicola* LW15^T^, respectively.

**TABLE 1:** Average nucleotide identity based on BLAST and *in silico* DNA-DNA hybridization values

Acinetobacter species and strain	Accession no.	ANI (%)/*is*DDH (%)^*a*^ of:		GC content (%)
		WCHAc060012^T^	WCHAc060115^T^	
*A. albensis* ANC 4874^T^	FMBK00000000.1	79.15/20.2	79.48/20.9	38.4
*A. apis* ANC 5114^T^	FZLN00000000.1	77.71/20.3	77.99/20.0	38.3
A. baumannii ATCC 19606^T^	APRG00000000.1	78.75/19.9	79.37/21.1	39.1
A. baylyi CIP 107474^T^	APPT00000000.1	78.47/19.7	78.91/20.7	40.4
*A. beijerinckii* CIP 110307^T^	APQL00000000.1	78.46/20.8	79.42/21.1	38.3
*A. bereziniae* CIP 70.12^T^	APQG00000000.1	79.26/21.1	82.98/27.1	38.2
*A. bohemicus* ANC 3994^T^	APOH00000000.1	79.70/21.1	80.04/21.8	39.6
*A. boissieri* ANC 4422^T^	FMYL00000000.1	77.81/19.5	77.79/20.1	38.0
*A. bouvetii* CIP 107468^T^	APQD00000000.1	81.52/22.4	79.36/20.7	45.0
*A. brisouii* CIP 110357^T^	AYEU00000000.1	78.70/21.7	79.13/22.3	41.7
A. calcoaceticus DSM 30006^T^	APQI00000000.1	78.82/20.2	78.99/21.4	38.6
*A. celticus* ANC 4603^T^	MBDL00000000.1	80.30/20.8	79.77/21.0	39.3
*A. chengduensis* WCHAc060005^T^	RCHC00000000.1	81.99/22.2	79.52/21.8	39.9
*A. chinensis* WCHAc010005^T^	CP032134.1	79.78/21.0	80.09/22.9	42.4
*A. colistiniresistens* NIPH 2036^T^	ATGK00000000.1	78.72/20.5	81.37/28.6	41.0
*A. courvalinii* ANC 3623^T^	APSA00000000.1	78.74/20.6	79.00/21.0	42.0
*A. cumulans* WCHAc060092^T^	PYIW00000000.1	82.70/23.4	79.56/21.9	40.2
*A. defluvii* WCHA30^T^	MAUF00000000.1	80.03/21.7	83.08/27.6	38.0
*A. dispersus* ANC 4105^T^	APRL00000000.1	78.86/20.2	79.19/21.3	40.4
*A. equi* 114^T^	CP012808.1	79.94/21.4	79.86/21.9	34.9
*A. gandensis* ANC 4275^T^	LZDS00000000.1	81.09/21.4	79.94/21.4	39.7
*A. gerneri* CIP 107464^T^	APPN00000000.1	79.48/22.5	80.13/22.9	37.7
*A. guerrae* AC 1271^T^	LXGN00000000.1	78.65/19.2	78.89/20.1	39.2
*A. guillouiae* CIP 63.46^T^	APOS00000000.1	79.10/21.3	82.02/24.6	38.2
*A. gyllenbergii* CIP 110306^T^	ATGG00000000.1	78.52/20.1	79.35/22.5	40.8
*A. haemolyticus* CIP 64.3^T^	APQQ00000000.1	78.95/21.5	79.22/22.3	39.7
*A. halotolerans* JCM 31009^T^	SGIM00000000.1	78.58/19.8	79.02/20.5	40.0
*A. harbinensis* HITLi7^T^	JXBK00000000.1	79.01/19.9	79.18/21.1	40.9
*A. indicus* CIP 110367^T^	AYET00000000.1	79.99/21.3	79.69/21.5	45.4
*A. johnsonii* CIP 64.6^T^	APON00000000.1	80.58/21.6	80.03/22.6	41.5
A. junii CIP 107470^T^	APPS01000079.1	79.07/21.1	79.11/21.6	38.8
*A. kookii* ANC 4667^T^	FMYO00000000.1	80.35/21.2	79.78/20.9	43.0
*A. lactucae* NRRL B-41902^T^	LRPE00000000.1	78.85/19.8	78.98/21.3	38.6
*A. lanii* 185^T^	CP049916.1	79.66/22.2	79.76/21.8	41.3
*A. larvae* BRTC-1^T^	CP016895.1	78.06/20.6	78.21/21.8	41.6
A. lwoffii NIPH 512^T^	AYHO00000000.1	80.01/21.3	79.12/22.0	43.0
*A. modestus* NIPH 236^T^	APOJ00000000.1	78.72/20.3	79.53/21.9	38.4
*A. nectaris* CIP 110549^T^	AYER00000000.1	77.98/20.1	78.08/20.5	36.7
A. nosocomialis NIPH 2119^T^	APOP00000000.1	78.72/20.0	79.22/21.4	38.7
*A. parvus* CIP 108168^T^	APOM00000000.1	79.12/21.8	79.18/22.3	41.7
*A. piscicola* KCTC 62134^T^	NIFO00000000.1	79.33/21.0	89.69/36.4	37.2
A. pittii ATCC 19004^T^	APQP01000014.1	78.73/20.2	78.98/21.2	38.8
*A. populi* PBJ7^T^	NEXX00000000.1	77.54/20.6	78.02/21.1	40.2
*A. portensis* AC 877^T^	LWRV00000000.1	80.10/21.2	80.14/21.8	36.6
*A. pragensis* ANC 4149^T^	LUAW00000000.1	81.32/22.1	79.09/20.5	44.0
*A. proteolyticus* NIPH 809^T^	APOI00000000.1	78.57/19.9	79.80/22.2	41.1
*A. pseudolwoffii* ANC 5044^T^	PHRG00000000.1	80.14/21.0	79.42/21.3	43.3
*A. pullicarnis* S23^T^	VCMZ00000000.1	78.10/21.7	79.92/24.9	41.5
*A. pullorum* B301^T^	JAAARQ000000000.1	80.21/21.5	80.14/22.3	37.0
*A. puyangensis* ANC 4466^T^	OANT00000000.1	77.09/20.1	77.71/20.5	40.2
*A. qingfengensis* ANC 4671^T^	MKKK00000000.1	77.52/19.9	77.88/21.0	38.1
*A. radioresistens* DSM 6976^T^	APQF00000000.1	78.59/19.8	78.78/20.8	41.7
*A. rongchengensis* WCHAc060115^T^	RAXT00000000.1	79.46/21.7		37.7
*A. rudis* CIP 110305^T^	ATGI00000000.1	78.27/20.8	78.90/21.0	39.0
*A. schindleri* CIP 107287^T^	APPQ00000000.1	80.38/21.4	79.65/21.7	42.2
*A. seifertii* NIPH 973^T^	APOO00000000.1	78.94/20.7	79.35/22.6	38.6
*A. shaoyimingii* 323-1^T^	CP049801.1	79.61/22.3	79.92/21.7	38.3
*A. sichuanensis* WCHAc060041^T^	PYIX00000000.1	79.86/21.9	83.12/27.2	37.2
*A. soli* CIP 110264^T^	APPU00000000.1	78.28/19.7	78.64/20.2	43.2
*A. tandoii* CIP 107469^T^	AQFM00000000.1	79.89/20.5	80.58/23.2	40.0
*A. tianfuensis* WCHAc060012^T^	RAXV00000000.1		79.46/21.7	42.3
*A. tjernbergiae* CIP 107465^T^	AYEV00000000.1	78.79/20.1	79.63/22.0	38.5
*A. towneri* CIP 107472^T^	APPY00000000.1	80.03/21.7	79.97/21.9	41.2
*A. ursingii* CIP 107286^T^	APQA00000000.1	78.73/19.9	79.25/22.1	40.1
*A. variabilis* NIPH 2171^T^	APRS00000000.1	79.95/20.9	79.76/22.4	42.0
*A. venetianus* CIP 110063^T^	APPO00000000.1	78.77/20.4	79.11/20.8	39.1
*A. vivianii* NIPH 2168^T^	APRW00000000.1	78.90/20.6	79.20/21.3	41.4
*A. wanghuae* dk386^T^	CP045650.1	79.88/21.0	79.65/21.0	40.6
*A. wuhouensis* WCHA60^T^	MBPR00000000.1	79.95/22.1	82.04/24.0	38.1

a ANI and *is*DDH values were calculated using fastANI v1.32 (46) and the genome-to-genome distance calculator (formula 2) (47), respectively.

After phenotypic characterizations (see below), we propose strain WCHAc060012^T^ with the name Acinetobacter *tianfuensis* sp. nov. (tian.fu.en’sis. N.L. masc. adj. tianfuensis, referring to Chengdu City, Sichuan Province, China) and WCHAc060115^T^ with the name Acinetobacter *rongchengensis* sp. nov. (rong.cheng.en’sis. N.L. masc. adj. rongchengensis, another name referring to Chengdu City, Sichuan Province, China). The type strain of Acinetobacter *tianfuensis* and Acinetobacter *rongchengensis* is WCHAc060012^T^ (=GDMCC 1.1623^T^ =JCM 33510^T^) and WCHAc060115^T^ (=GDMCC 1.1625^T^ =JCM 33512^T^), respectively.

### The two novel Acinetobacter species may be able to be differentiated from other Acinetobacter species by a combination of phenotypic characteristics.

The phenotypic characteristics tested using the genus-targeted set of physiological and metabolic tests are presented in the standard way used in previous nomenclatural proposals (32, 33). The phenotypes for the two novel Acinetobacter species, together with those for all known Acinetobacter species with validly published names, are summarized in Data Set S1 in the supplemental material. For both strains, growth occurs at various pHs from 7 to 8 and the temperatures range 20 to 35°C. Strain WCHAc060012^T^ grows at 30°C in the presence of 0% to 3% (wt/vol) NaCl in tryptic soy broth (TSB), while WCHAc060115^T^ grows in 0% to 4% (wt/vol) NaCl. Both strains were positive for the catalase test but negative for the oxidase activity. Cells of the two strains are Gram-negative coccobacilli; strictly aerobic; nonsporogenous; incapable of swimming motility; and capable of growing on media such as tryptic soy agar (TSA), Luria-Bertani (LB) agar, BHI agar, and Müller-Hinton agar (all from Hopebio). Colonies are light yellow, circular, opaque, smooth, convex, with entire margins, and approximately 1.0 to 2.0 mm in diameter after 24 h of incubation at 30°C on BHI agar plates.

10.1128/mSystems.00237-21.6 DATA SET S1 Phenotypic properties of *A. tianfuensis* sp. nov., *A. rongchengensis* sp. nov. and the Acinetobacter species with validly published names. Download Data Set S1, XLSX file, 0.03 MB. Copyright © 2021 Qin et al. 2021 Qin et al. https://creativecommons.org/licenses/by/4.0/ This content is distributed under the terms of the Creative Commons Attribution 4.0 International license.

Phenotypic differences between the two novel Acinetobacter species and each of the known species with validly published names are indicated in Data Set S1. When considering only clearly positive or clearly negative results, the most useful combinations of characteristics for differentiating WCHAc060012^T^ from all known Acinetobacter species include growth on l-glutamate, d-malate, malonate, and phenylacetate but no growth on l-arabinose, l-arginine, azelate, and glutarate (Data Set S1). Strain WCHAc060115^T^ could be differentiated from all known Acinetobacter species by the combination of assimilation *trans*-aconitate, citrate (Simmons’), and l-tartrate but not β-alanine and 4-aminobutyrate (Data Set S1).

We also identified antimicrobial resistance genes from genome sequences of the two strains (see Table S1 in the supplemental material). Both strains had genes mediating resistance to aminoglycosides, sulfonamides, and macrolides, while WCHAc060115^T^ also harbored two carbapenemase genes, namely, *bla*_NDM-1_ and *bla*_OXA-58_, and WCHAc060012^T^ carried a tetracycline-resistant gene *tet*(39).

10.1128/mSystems.00237-21.3 TABLE S1 Antimicrobial resistance genes of *A. tianfuensis* WCHAc060012^T^ and *A. rongchengensis* WCHAc060115^T^. Download Table S1, PDF file, 0.1 MB. Copyright © 2021 Qin et al. 2021 Qin et al. https://creativecommons.org/licenses/by/4.0/ This content is distributed under the terms of the Creative Commons Attribution 4.0 International license.

### Acinetobacter *pullorum* Elnar et al. 2020 and Acinetobacter *portensis* Ana et al. 2020 are the same species.

During the process of studying WCHAc060012^T^ and WCHAc060115^T^, we also found a pair of synonyms, namely, *A. pullorum* and *A. portensis*. *A. pullorum* B301^T^ was isolated from raw chicken meat at a local market in Korea (14). It has been shown that *A. pullorum* B301^T^ is closely related to Acinetobacter celticus ANC 4603^T^ (14). Four *A. portensis* strains were isolated from raw meat samples in supermarkets in Porto, Portugal. *A. portensis* is also closely related to *A. celticus* ANC 4603^T^ (15). A comparison of the 16S rRNA gene sequences for the two type strains showed a 99.70% similarity. The draft genome sequence of *A. pullorum* B301^T^ (GenBank accession no. JAAARQ000000000) and that of *A. portensis* AC 877^T^ (GenBank accession no. LWRV00000000) have a 90.4% *is*DDH value and a 98.59% ANI value. Both ANI and *is*DDH analyses clearly indicate that the two species are actually the same species. In the phylogenomic tree, *A. pullorum* B301^T^ and *A. portensis* AC 877^T^ indeed cluster together (Fig. 1, highlighted in red).

A comparison of the physiological and biochemical features of the two type strains shows phenotype coherence, which is summarized in Table S2 in the supplemental material. According to previous reports (14), *A. pullorum* and *A. portensis* are different in the acidification of d-glucose and utilization of β-alanine and d-glucose, which is likely due to intraspecies variability or assay conditions. Based on principles by the International Code of Nomenclature of Bacteria (12), *A. pullorum* has the priority of species name over *A. portensis*. We therefore propose that *A. portensis* (15) is a later heterotypic synonym of *A. pullorum* (14).

10.1128/mSystems.00237-21.4 TABLE S2 The characteristics of strains of *A. pullorum* and *A. portensis*. Download Table S2, PDF file, 0.1 MB. Copyright © 2021 Qin et al. 2021 Qin et al. https://creativecommons.org/licenses/by/4.0/ This content is distributed under the terms of the Creative Commons Attribution 4.0 International license.

### Curation of Acinetobacter genomes with the updated taxonomy.

Based on the above findings, the valid species names of Acinetobacter should be updated to comprise 68 species at present (Table 2). In addition, there are 20 tentative species designations of Acinetobacter (www.szu.cz/anemec/Classification.pdf) (Table 2). We then applied the updated Acinetobacter taxonomy to curate the 5,997 Acinetobacter strains with genome sequences deposited in GenBank (accessed by 1 August 2020). Before curation, we performed a quality-control check for all of the genomes. Among the 5,997 genomes, 2,041 were discarded due to low quality defined by >300 contigs for individual genomes (*n* = 444), a <50-kb *N*_50_ value (*n* = 458), <90% genome completeness (*n* = 20), genome contamination (*n* = 206), or genome heterogeneity (*n* = 913). We then used the remaining 3,956 genomes for precise species identification by both ANI and *is*DDH. Among the 3,956 Acinetobacter genomes, 3,777 were labeled with a known Acinetobacter species name (Data Set S2). The remaining 179 strains were labeled only with Acinetobacter sp. (*n* = 175), Acinetobacter genomosp. (*n* = 2), Acinetobacter calcoaceticus*/*Acinetobacter baumannii complex (*n* = 1), or uncultured Acinetobacter (*n* = 1) (Data Set S2), which were updated by our curation. Species were misidentified for 55 Acinetobacter genomes (Data Set S2 and summarized in Table S3 in the supplemental material). The 55 misidentified genomes include 13 labeled with A. baumannii but actually belonging to other Acinetobacter species and four of non-A. baumannii Acinetobacter species actually belonging to other closely related species (Table S3), while the remaining 38 genomes should be assigned to novel taxa (see below for details). Therefore, there were 234 genomes whose species identification needs to be corrected (*n* = 55) or updated (*n* = 179) according to the findings in this study (Data Set S2).

**TABLE 2:** Updated classification and nomenclature of the genus Acinetobacter before species curation for genomes in GenBank

Species name	Type strain or reference strain	Accession no.
Valid (*n* = 68)		
Acinetobacter albensis	ANC 4874^T^	FMBK00000000
Acinetobacter apis	ANC 5114^T^	FZLN00000000
Acinetobacter baumannii	CIP 70.34^T^	APRG00000000
Acinetobacter baylyi	CIP 107474^T^	APPT00000000
Acinetobacter beijerinckii	CIP 110307^T^	APQL00000000
Acinetobacter bereziniae	CIP 70.12^T^	APQG00000000
Acinetobacter bohemicus^*a*^	ANC 3994^T^	APOH00000000
Acinetobacter boissieri	ANC 4422^T^	FMYL00000000
Acinetobacter bouvetii	CIP 107468^T^	APQD00000000
Acinetobacter brisouii	ANC 4119^T^	APPR00000000
Acinetobacter calcoaceticus	CIP 81.8^T^	APQI00000000
Acinetobacter celticus	ANC 4603^T^	MBDL00000000
Acinetobacter *chengduensis*	WCHAc060005^T^	RCHC00000000
Acinetobacter *chinensis*	WCHAc010005^T^	CP032134
Acinetobacter colistiniresistens	NIPH 2036^T^	ATGK00000000
Acinetobacter courvalinii	ANC 3623^T^	APSA00000000
Acinetobacter *cumulans*	WCHAc060092^T^	PYIW00000000
Acinetobacter defluvii	WCHA30^T^	CP029397
Acinetobacter dispersus	ANC 4105^T^	APRL00000000
Acinetobacter equi	114^T^	CP012808
Acinetobacter gandensis	ANC 4275^T^	LZDS00000000
Acinetobacter gerneri	CIP 107464^T^	APPN00000000
Acinetobacter *guerrae*	AC 1271^T^	LXGN00000000
Acinetobacter guillouiae	CIP 63.46^T^	APOS00000000
Acinetobacter gyllenbergii	CIP 110306^T^	ATGG00000000
Acinetobacter haemolyticus	CIP 64.3^T^	APQQ00000000
Acinetobacter halotolerans	JCM 31009^T^	SGIM00000000
Acinetobacter harbinensis	HITLi7^T^	JXBK00000000
Acinetobacter indicus^*b*^	ANC 4215^T^	ATGH00000000
Acinetobacter johnsonii	CIP 64.6^T^	APON00000000
Acinetobacter junii^*c*^	CIP 64.5^T^	APPX00000000
Acinetobacter kookii	ANC 4667^T^	FMYO00000000
Acinetobacter lactucae^*d*^	NRRL B-41902^T^	LRPE00000000
Acinetobacter *lanii*	185^T^	CP049916
Acinetobacter larvae	BRTC-1^T^	CP016895
Acinetobacter lwoffii^*e*^	NIPH 512^T^	AYHO00000000
Acinetobacter modestus	NIPH 236^T^	APOJ00000000
Acinetobacter nectaris	CIP 110549^T^	AYER00000000
Acinetobacter nosocomialis	NIPH 2119^T^	APOP00000000
Acinetobacter parvus	CIP 108168^T^	APOM00000000
Acinetobacter *piscicola*	LW15^T^	NIFO00000000
Acinetobacter pittii	CIP 70.29^T^	APQP00000000
Acinetobacter populi	PBJ7^T^	NEXX00000000
Acinetobacter pragensis	ANC 4149^T^	LUAW00000000
Acinetobacter proteolyticus	NIPH 809^T^	APOI00000000
Acinetobacter *pseudolwoffii*	ANC 5044^T^	PHRG00000000
Acinetobacter *pullicarnis*	S23^T^	CP036789
Acinetobacter *pullorum*^*f*^	B301^T^	JAAARQ000000000
Acinetobacter puyangensis	ANC 4466^T^	OANT00000000
Acinetobacter qingfengensis	ANC 4671^T^	MKKK00000000
Acinetobacter radioresistens	CIP 103788^T^	APQF00000000
Acinetobacter *rongchengensis*	WCHAc060115^T^	RAXT00000000
Acinetobacter rudis	CIP 110305^T^	ATGI00000000
Acinetobacter schindleri	CIP 107287^T^	APPQ00000000
Acinetobacter seifertii	NIPH 973^T^	APOO00000000
Acinetobacter *shaoyimingii*	323-1^T^	CP049801
Acinetobacter *sichuanensis*	WCHAc060041^T^	PYIX00000000
Acinetobacter soli	CIP 110264^T^	APPU00000000
Acinetobacter tandoii	CIP 107469^T^	AQFM00000000
Acinetobacter *tianfuensis*	WCHAc060012^T^	RAXV00000000
Acinetobacter tjernbergiae	CIP 107465^T^	AYEV00000000
Acinetobacter towneri	CIP 107472^T^	APPY00000000
Acinetobacter ursingii	CIP 107286^T^	APQA00000000
Acinetobacter variabilis	NIPH 2171^T^	APRS00000000
Acinetobacter venetianus	CIP 110063^T^	APPO00000000
Acinetobacter vivianii	NIPH 2168^T^	APRW00000000
Acinetobacter *wanghuae*	dk386^T^	CP045650
Acinetobacter *wuhouensis*	WCHA60^T^	CP031716
Tentative designations (*n* = 20)		
Acinetobacter *kyonggiensis*	ANC 5109	FNPK00000000
Acinetobacter *marinus*	ANC 3699	FMYK00000000
Acinetobacter *oleivorans*	DR1	CP002080
Genomic sp. 6	CIP a165	APOK00000000
Genomic sp. 15BJ	CIP 110321	AQFL00000000
Genomic sp. 16	CIP 70.18	APRN00000000
Taxon 21	ANC 3929	APRH00000000
Taxon 22	NIPH 2100	APSB00000000
Taxon 24A	ANC 4655	NEGF00000000
Taxon 24B	ANC 4471	SJNZ00000000
Taxon 25A	ANC 3789	APOY00000000
Taxon 25B	ANC 4633	SJNX00000000
Taxon 27	ANC 4169	NEGE00000000
Taxon 32	ANC 4218	NEGD00000000
Taxon 34	ANC 4470	NEGC00000000
Taxon 35	ANC 4999	NEGB00000000
Taxon 36	ANC 4945	MVKX00000000
Taxon 37	WCHAc010034	CP032279
Taxon 38	ANC 3903	NEGA00000000
Taxon 39	ANC 4204	NEFZ00000000

a Acinetobacter pakistanensis is a later synonym of Acinetobacter bohemicus (57).

b Acinetobacter guangdongensis is a later synonym of Acinetobacter indicus (20).

c Acinetobacter grimontii is a later synonym of Acinetobacter junii (21).

d Acinetobacter dijkshoorniae is a later synonym of Acinetobacter lactucae (22).

e Acinetobacter mesopotamicus is a later synonym of Acinetobacter lwoffii (19).

f *Acinetobacter portensis* is a later synonym of Acinetobacter *pullorum* (this study).

10.1128/mSystems.00237-21.5 TABLE S3 The 55 Acinetobacter genome sequences with misidentified species in NCBI. Download Table S3, PDF file, 0.1 MB. Copyright © 2021 Qin et al. 2021 Qin et al. https://creativecommons.org/licenses/by/4.0/ This content is distributed under the terms of the Creative Commons Attribution 4.0 International license.

10.1128/mSystems.00237-21.7 DATA SET S2 The 3,956 Acinetobacter genome sequences available in GenBank (accessed by 1 August 2020). Download Data Set S2, XLSX file, 0.4 MB. Copyright © 2021 Qin et al. 2021 Qin et al. https://creativecommons.org/licenses/by/4.0/ This content is distributed under the terms of the Creative Commons Attribution 4.0 International license.

**FIG 2:**
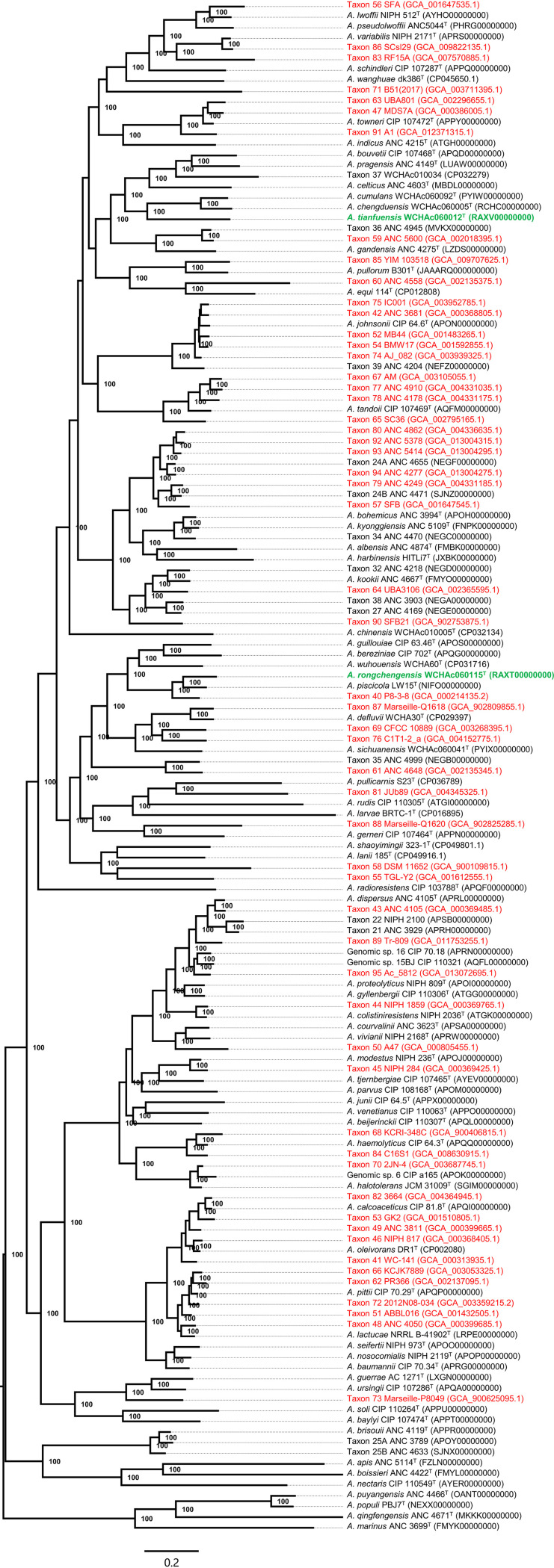
Phylogenomic tree of Acinetobacter species with validly published names and tentative taxa. The phylogenomic tree was inferred based on the alignment of 1,397 core genes. Strains and their nucleotide accession no. are listed alongside the names of species, and 100% bootstrap are shown. Bar, value indicates the nucleotide substitutions per site. The two novel Acinetobacter species are depicted in green, while novel Acinetobacter taxa identified in this study, namely, taxon 40 to 95, are in red.

After precise species identification, among the 3,956 Acinetobacter strains with genome sequences available, most (*n* = 3,124, 79.0%) belonged to A. baumannii, followed by Acinetobacter pittii (*n* = 174, 4.4%), Acinetobacter nosocomialis (*n* = 103, 2.6%), and Acinetobacter indicus (*n* = 68, 1.7%; Table 3). However, 94 (2.4%) strains could not be assigned to any known Acinetobacter species nor to any known tentative species designations (Data Set S3). Instead, the 94 strains could be assigned to 56 potentially novel Acinetobacter species, which are named taxon 40 to 95 here (Table 4 and Fig. 2), as Acinetobacter taxon 39 has been used before. Characterization of taxon 40 to 95 by phenotype methods is warranted to further establish their species status with proper species names under current International Code of Nomenclature of Prokaryotes (12).

**TABLE 3:** Species distribution of 3,956 Acinetobacter strains with genome sequences available in GenBank

Species	No. of genomes	Taxon without a species name^*a*^	No. of genomes
Acinetobacter albensis	2	Genomic sp. 6	2
Acinetobacter apis	1	Genomic sp. 15BJ	1
Acinetobacter baumannii	3,124	Genomic sp. 16	6
Acinetobacter baylyi	11	Taxon 21	1
Acinetobacter beijerinckii	3	Taxon 22	1
Acinetobacter bereziniae	1	Taxon 24A	1
Acinetobacter bohemicus	1	Taxon 24B	3
Acinetobacter boissieri	1	Taxon 25A	2
Acinetobacter bouvetii	3	Taxon 25B	2
Acinetobacter brisouii	4	Taxon 27	1
Acinetobacter calcoaceticus	15	Taxon 32	1
Acinetobacter celticus	1	Taxon 34	1
Acinetobacter *chengduensis*	2	Taxon 35	1
Acinetobacter *chinensis*	2	Taxon 36	1
Acinetobacter colistiniresistens	4	Taxon 37	1
Acinetobacter courvalinii	5	Taxon 38	1
Acinetobacter *cumulans*	8	Taxon 39	2
Acinetobacter defluvii	2	**Taxon 40**	1
Acinetobacter dispersus	1	**Taxon 41**	1
Acinetobacter equi	1	**Taxon 42**	3
Acinetobacter gandensis	1	**Taxon 43**	2
Acinetobacter gerneri	2	**Taxon 44**	2
Acinetobacter *guerrae*	3	**Taxon 45**	5
Acinetobacter guillouiae	1	**Taxon 46**	4
Acinetobacter gyllenbergii	4	**Taxon 47**	2
Acinetobacter haemolyticus	20	**Taxon 48**	1
Acinetobacter halotolerans	1	**Taxon 49**	1
Acinetobacter harbinensis	1	**Taxon 50**	1
Acinetobacter indicus	68	**Taxon 51**	1
Acinetobacter johnsonii	8	**Taxon 52**	7
Acinetobacter junii	27	**Taxon 53**	2
Acinetobacter kookii	2	**Taxon 54**	5
Acinetobacter *kyonggiensis*	1	**Taxon 55**	1
Acinetobacter lactucae	7	**Taxon 56**	2
Acinetobacter *lanii*	2	**Taxon 57**	1
Acinetobacter larvae	1	**Taxon 58**	1
Acinetobacter lwoffii	17	**Taxon 59**	2
Acinetobacter *marinus*	1	**Taxon 60**	1
Acinetobacter modestus	2	**Taxon 61**	1
Acinetobacter nectaris	1	**Taxon 62**	3
Acinetobacter nosocomialis	103	**Taxon 63**	1
Acinetobacter *oleivorans*	7	**Taxon 64**	1
Acinetobacter parvus	8	**Taxon 65**	3
Acinetobacter *piscicola*	1	**Taxon 66**	4
Acinetobacter pittii	174	**Taxon 67**	1
Acinetobacter populi	1	**Taxon 68**	1
Acinetobacter pragensis	1	**Taxon 69**	1
Acinetobacter proteolyticus	5	**Taxon 70**	1
Acinetobacter *pseudolwoffii*	3	**Taxon 71**	3
Acinetobacter *pullicarnis*	1	**Taxon 72**	1
Acinetobacter *pullorum*	2	**Taxon 73**	1
Acinetobacter puyangensis	1	**Taxon 74**	1
Acinetobacter qingfengensis	2	**Taxon 75**	1
Acinetobacter radioresistens	32	**Taxon 76**	2
Acinetobacter *rongchengensis*	1	**Taxon 77**	1
Acinetobacter rudis	1	**Taxon 78**	1
Acinetobacter schindleri	10	**Taxon 79**	1
Acinetobacter seifertii	19	**Taxon 80**	1
Acinetobacter *shaoyimingii*	2	**Taxon 81**	1
Acinetobacter *sichuanensis*	1	**Taxon 82**	1
Acinetobacter soli	22	**Taxon 83**	3
Acinetobacter tandoii	2	**Taxon 84**	1
Acinetobacter *tianfuensis*	1	**Taxon 85**	1
Acinetobacter tjernbergiae	3	**Taxon 86**	2
Acinetobacter towneri	11	**Taxon 87**	1
Acinetobacter ursingii	29	**Taxon 88**	1
Acinetobacter variabilis	6	**Taxon 89**	1
Acinetobacter venetianus	10	**Taxon 90**	1
Acinetobacter vivianii	5	**Taxon 91**	1
Acinetobacter *wanghuae*	2	**Taxon 92**	1
Acinetobacter *wuhouensis*	6	**Taxon 93**	1
		**Taxon 94**	1
		**Taxon 95**	1

a Taxa identified in this study are highlighted in bold.

**TABLE 4:** Tentative taxon assignations for novel, unnamed Acinetobacter species identified in this study

Taxon	Accession no.	Reference strain^*a*^	Closest species or taxon	ANI (%)	*is*DDH (%)
40	GCA_000214135.2	P8-3-8	*A. piscicola*	88.77	34.4
41	GCA_000313935.1	WC-141	*A. oleivorans*	93.08	49.3
42	GCA_000368805.1	ANC 3681	*A. johnsonii*	95.83	66.6
43	GCA_000369485.1	ANC 4105	*A. dispersus*	95.61	62.4
44	GCA_000369765.1	NIPH 1859	*A. colistiniresistens*	95.29	60.3
45	GCA_000369425.1	NIPH 284	*A. modestus*	94.51	54.8
46	GCA_000368405.1	NIPH 817	*A. oleivorans*	95.2	61.3
47	GCA_000386005.1	MDS7A	*A. towneri*	93.8	52.9
48	GCA_000399685.1	ANC 4050	*A. lactucae*	94.01	53.5
49	GCA_000399665.1	ANC 3811	*A. oleivorans*	94.28	55.8
50	GCA_000805455.1	A47	*A. courvalinii*	88	31.9
51	GCA_001432505.1	ABBL016	A. pittii	94.84	58
52	GCA_001483265.1	MB44	*A. johnsonii*	95.84	65.6
53	GCA_001510805.1	GK2	A. calcoaceticus	93.65	51.5
54	GCA_001592855.1	BMW17	*A. johnsonii*	95.6	64.9
55	GCA_001612555.1	TGL-Y2	*A. bohemicus*	80.2	22.1
56	GCA_001647535.1	SFA	A. lwoffii	90.48	38.1
57	GCA_001647545.1	SFB	Taxon 24B	89.6	36.8
58	GCA_900109815.1	DSM 11652	*A. cumulans*	80.48	21.5
59	GCA_002018395.1	ANC 5600	Taxon 36	95.48	62.6
60	GCA_002135375.1	ANC 4558	*A. equi*	81.73	23
61	GCA_002135345.1	ANC 4648	Taxon 35	87.82	33
62	GCA_002137095.1	PR366	A. pittii	95.08	59.4
63	GCA_002296655.1	UBA801	*A. towneri*	93.42	50.5
64	GCA_002365595.1	UBA3106	*A. kookii*	88.29	32.6
65	GCA_002795165.1	SC36	*A. tandoii*	87.13	29.9
66	GCA_003053325.1	KCJK7889	A. pittii	95.5	62.5
67	GCA_003105055.1	AM	*A. tandoii*	92	43.2
68	GCA_900406815.1	KCRI-348C	*A. haemolyticus*	92.5	46.7
69	GCA_003268395.1	CFCC 10889	*A. wuhouensis*	85.29	29.1
70	GCA_003687745.1	2JN-4	*A. halotolerans*	95.44	59.7
71	GCA_003711395.1	B51(2017)	*A. gandensis*	80.94	21.9
72	GCA_003359215.2	2012N08-034	A. pittii	95.95	65.4
73	GCA_900625095.1	Marseille-P8049	*A. ursingii*	84.88	26.8
74	GCA_003939325.1	AJ_082	*A. johnsonii*	95.75	65.9
75	GCA_003952785.1	IC001	*A. johnsonii*	95.86	66.6
76	GCA_004152775.1	C1T1-2_a	*A. sichuanensis*	86.05	28.5
77	GCA_004331035.1	ANC 4910	*A. tandoii*	91	40
78	GCA_004331175.1	ANC 4178	*A. tandoii*	91.25	40.6
79	GCA_004331185.1	ANC 4249	Taxon 24B	95.48	61
80	GCA_004336635.1	ANC 4862	Taxon 24A	92.69	47.2
81	GCA_004345325.1	JUb89	*A. pullicarnis*	79.81	21.2
82	GCA_004364945.1	3664	A. calcoaceticus	95.97	65.8
83	GCA_007570885.1	RF15A	*A. variabilis*	83.02	24.6
84	GCA_008630915.1	C16S1	*A. haemolyticus*	93.7	50.1
85	GCA_009707625.1	YIM 103518	*A. pullorum*	87.32	30.6
86	GCA_009822135.1	SCsl29	*A. variabilis*	95.33	62.9
87	GCA_902809855.1	Marseille-Q1618	*A. defluvii*	91.33	42.3
88	GCA_902825285.1	Marseille-Q1620	*A. gerneri*	81.13	22.2
89	GCA_011753255.1	Tr-809	*A. dispersus*	91.34	41.3
90	GCA_902753875.1	SFB21	Taxon 32	85.48	27.8
91	GCA_012371315.1	A1	*A. towneri*	88.81	32.8
92	GCA_013004315.1	ANC 5378	Taxon 24A	92.8	47.2
93	GCA_013004295.1	ANC 5414	Taxon 24A	92.74	47
94	GCA_013004275.1	ANC 4277	Taxon 24A	95.98	64.1
95	GCA_013072695.1	Ac_5812	Genomic sp. 16	92.7	47.2

a The strain with genome sequence deposited in GenBank at the earliest date was selected as the reference strain for the newly identified taxa.

10.1128/mSystems.00237-21.8 DATA SET S3 The potential tentative species designation. Download Data Set S3, XLSX file, 0.02 MB. Copyright © 2021 Qin et al. 2021 Qin et al. https://creativecommons.org/licenses/by/4.0/ This content is distributed under the terms of the Creative Commons Attribution 4.0 International license.

### Acinetobacter is indeed a single genus comprising 144 species at present.

The identification of the 56 taxa also extends the number of Acinetobacter species to 144, including 68 with species names and 76 unnamed taxa. The large number of species raises the question whether Acinetobacter is indeed a single genus or actually should be divided into different genera. ANI values among type strains of all species and reference strains of all taxa of the genus Acinetobacter are ≥76.97% (76.97% to 95.98%) (see Data Set S4 in the supplemental material). The ANI values are higher than 72.50% to 73.70%, which has been proposed as the 95% confidence interval of the boundary to define a bacterial genus (34). To further verify the genus Acinetobacter, the average amino acid identity (AAI) values among type strains of all species and reference strains of all taxa of the genus Acinetobacter were also calculated, which are >66% (66.5% to 97.4%) (see Data Set S5 in the supplemental material). This is higher than the proposed cutoff of 65% AAI used to define a bacterial genus (34, 35). Both ANI and AAI analyses suggest that all Acinetobacter species and unnamed taxa identified so far indeed belong to a single genus.

10.1128/mSystems.00237-21.9 DATA SET S4 Average nucleotide identity between type strains of Acinetobacter species and reference strains of Acinetobacter taxa without species names. Download Data Set S4, XLSX file, 0.1 MB. Copyright © 2021 Qin et al. 2021 Qin et al. https://creativecommons.org/licenses/by/4.0/ This content is distributed under the terms of the Creative Commons Attribution 4.0 International license.

10.1128/mSystems.00237-21.10 DATA SET S5 Average amino acid identity between type strains of Acinetobacter species and reference strains of Acinetobacter taxa without species names. Download Data Set S5, XLSX file, 0.1 MB. Copyright © 2021 Qin et al. 2021 Qin et al. https://creativecommons.org/licenses/by/4.0/ This content is distributed under the terms of the Creative Commons Attribution 4.0 International license.

## DISCUSSION

In this study, we first found and characterized two novel Acinetobacter species. We also found that *A. pullorum* and *A. portensis* are synonyms and then updated the taxonomy of the genus Acinetobacter. We applied the updated taxonomic assignments to curate genome sequences deposited in GenBank with the label of Acinetobacter and found that 6% (*n* = 234) of the 3,956 genomes with good quality need to be corrected or updated for species identification. We also identified 56 previously unknown tentative species designations, which further update the genus Acinetobacter to comprise 144 species, including 68 with species names and 76 taxa without species names. Such a large number of species raises the question whether Acinetobacter should be divided into multiple genera. Although the boundary of bacterial genera based on genome sequences is less established than that of species and requires more studies (9), our ANI and AAI analyses suggest that all Acinetobacter species indeed belong to a single genus. The mechanisms and factors driving the divergence of Acinetobacter to form the evolutionary trajectory and generate the remarkable species diversity and form have not been understood (36, 37).

Along with many Acinetobacter species identified recently (11, 13–18), the above findings highlight that Acinetobacter is a highly diverse and complex group (38). The species status of two novel Acinetobacter species, namely, *A. tianfuensis* and *A. rongchengensis*, was established by both genome- and phenotype-based methods. In addition to known species, there were 76 tentative novel Acinetobacter taxa, including 56 identified in this study. The identification of new taxa invites more studies on these tentative species by both genome- and phenotype-based methods to establish their species status and to propose appropriate species names under the current code for prokaryotes (12). Alternatively, it has also been proposed to create a new code that would use DNA sequences as the type material to rule the nomenclature of prokaryotes (39) or to establish placeholder species names using genome-based taxonomy (10). Indeed, there is an urgent need to find a solution to deal with the exploration of new taxonomic findings generated by genome sequencing (40).

In conclusion, we characterized and reported two novel Acinetobacter species, namely, *A. tianfuensis* and *A. rongchengensis*. *A. tianfuensis* may be distinguished from all other Acinetobacter species by its ability to grow on l-glutamate, d-malate, malonate, and phenylacetate but not grow on l-arabinose, l-arginine, azelate, and glutarate. *A. rongchengensis* may be differentiated from all other Acinetobacter species by the combination of assimilation *trans*-aconitate, citrate (Simmons’), and l-tartrate but not β-alanine and 4-aminobutyrate. We also found that *A. portensis* is a later heterotypic synonym of *A. pullorum.* We demonstrated that some Acinetobacter genome sequences deposited in GenBank are required to be corrected and identified 56 novel tentative Acinetobacter taxa, which warrant further phenotype-based characterizations.

## MATERIALS AND METHODS

### Strains and preliminary species identification.

Hospital sewage (1 ml) was collected from the influx mainstream of the wastewater treatment plant at West China Hospital in June 2018, which was added in 10 ml nutrient broth (Oxoid, Basingstoke, UK) and was incubated overnight at 30°C with shaking. The culture suspension was diluted to 0.5 McFarland standard and was then further diluted to 1:100 with saline. A 100-μl aliquot was then streaked onto an Acinetobacter chromogenic agar plate (CHROMagar, Paris, France). The plate was then incubated at 30°C overnight. All isolates recovered from the plate were subjected to preliminary species identification by partial sequencing of the RNA polymerase β subunit-encoding *rpoB* gene using PCR and Sanger sequencing as described previously (5). Isolates with ≤98% identity of the 861-bp partial *rpoB* sequence (corresponding to nucleotide positions 2915 to 3775 of A. baumannii CIP 70.34^T^; accession no. DQ207471) to type strains of all known Acinetobacter species may belong to novel species and were characterized as described below. Two Acinetobacter isolates, namely, WCHAc060012^T^ and WCHAc060115^T^, were recovered from the plate and had ≤98% identity of the 861-bp partial *rpoB* sequence to type strains of all known Acinetobacter species.

### Analysis based on 16S rRNA and *rpoB* genes.

Boiled lysates were used as the PCR template, and PCR amplicons were sequenced using the Sanger method (26). The nearly complete 16S rRNA gene sequences of WCHAc060012^T^ and WCHAc060115^T^ were obtained using PCR with universal primers 27F and 1492R (25). The 16S rRNA gene sequences of type strains of each Acinetobacter species were retrieved from their depositions in GenBank or from their whole-genome sequences. The longest common fragments of the 16S rRNA gene sequences (1,352 bp) were aligned using MAFFT v7.471 (41), and a maximum likelihood phylogenetic tree (42) based on the 1,352-bp sequences was inferred using RAxML v8.2.12 (43) with the general time reversible (GTR) model.

To further investigate the taxonomic position of WCHAc060012^T^ and WCHAc060115^T^, 861-bp partial *rpoB* sequences of type strains of each Acinetobacter species were retrieved from their depositions in GenBank or from their whole-genome sequences. Sequence alignment and the construction of a maximum-likelihood phylogenetic tree were performed as described above.

### Whole-genome sequencing of the two strains.

Genomic DNA from an overnight culture of each of the two strains was prepared using the QIAamp DNA minikit (Qiagen, Hilden, Germany) and was then subjected to whole-genome sequencing using the HiSeq X10 sequencing platform (Illumina, San Diego, CA, USA) with an approximate 250× coverage. Reads were *de novo* assembled into contigs using the program SPAdes v3.15.1 (44). Potential contaminations of WCHAc060012^T^ and WCHAc060115^T^ genomes were checked using CheckM v1.1.3 (45). Antimicrobial resistance genes were identified from genome sequences using the ABRicate program (https://github.com/tseemann/abricate) to query the ResFinder database 4.1 (https://cge.cbs.dtu.dk/services/ResFinder/).

### Precise species identification and phylogenomic analysis of the two strains.

Whole-genome sequences of type strains of all Acinetobacter species (Data Set S2) were retrieved from the NCBI database. Genome sequences of WCHAc060012^T^ and WCHAc060115^T^ were compared with those of type strains of Acinetobacter species using the average nucleotide identity based on BLAST (ANI) and *in silico* DNA-DNA hybridization (*is*DDH). ANI and *is*DDH values were calculated using the fastANI v1.32 (46) and genome-to-genome distance calculator (formula 2) (47) with the recommended parameters and/or default settings, respectively. A ≥96% ANI (31) or ≥70.0% isDDH (31, 47) was used as the cutoff to define a bacterial species.

A core genome phylogenetic tree based on concatenated sequences of core genes was constructed as described previously (48). Prokka v1.14.5 (49) and Prodigal v2.6.3 (50) were used to annotate these genome sequences, and protein-encoding sequences for each genome were retrieved for gene alignment and clustering using PIRATE v1.0.4 (51). The gene sequences were aligned and concatenated using MAFFT v7.471 (41) and AMAS v0.98 (52), which were then used to infer a phylogenomic tree using RAxML v8.2.12 (43) with GTR model plus gamma distribution and a 1,000-bootstrap test. The phylogenetic tree was visualized with FigTree (https://github.com/rambaut/figtree).

### Phenotypic characterization for strains of two novel species.

The metabolic and physiological properties were assessed using the standardized genus-targeted set of metabolic/physiological tests as described previously (5, 32, 53). The two strains were grown on brain heart infusion (BHI) agar (Oxoid) plates at 30°C overnight, and the colony morphology was observed by naked eyes. Cell morphology was visualized by light microscopy (CX21 microscope; Olympus, Japan). The Gram staining was carried out with a Gram staining kit (bioMérieux, Marcy l'Etoile, France). The cultivation temperature was 30°C unless indicated otherwise. Cell motility was tested in LB medium with 0.4% agar. Growth at various temperatures (20, 25, 32, 35, 37, 41, and 44°C) was tested in 5-ml aliquots of BHI broth dispensed into tubes (16-mm inner diameter) as described previously (5). Salt tolerance tests at different NaCl concentrations (0% to 10%, wt/vol, in increments of 1.0%) were performed in tryptic soy broth (TSB; Hopebio, Qingdao, China) after incubation for 2 days. Growth at pH 4.0 to 11.0 (at intervals of 1 pH unit, adjusted by adding HCl or NaOH) was examined in TSB for 2 days. The anaerobic growth was examined on a BHI agar plate, which was placed in an anaerobic bag (bioMérieux) at 30°C for 7 day (26). Aerobic acid production from glucose and gelatin hydrolysis was performed using the API 20NE system (bioMérieux), and the results were observed after 48 h. Hemolysis of sheep blood and utilization of citrate (Simmons’) were examined according to methods described previously (5). The characteristics for the assimilation of the other carbon sources were determined using the basal mineral medium (54) supplemented with 0.1% (wt/vol) carbon source as described previously (5).

### Curation of all available Acinetobacter genomes for precise species identification.

All genome sequences labeled as Acinetobacter species in GenBank (*n* = 5,997, accessed by 1 August 2020) were retrieved. The assemblies, completeness, contamination, and heterogeneity of the genomes were evaluated using QUAST v5.0.2 (55) and CheckM v1.1.3 (45). Genome assemblies were discarded due to low quality defined by >300 contigs, a <50-kb *N*_50_ value, <90% genome completeness, genome contamination indicated by ≥2 in CheckM, or none-zero genome heterogeneity value for individual genomes. ANI and *is*DDH values between each of the genomes and type strains of Acinetobacter genomes were calculated, using the fastANI v1.32 (46) and genome-to-genome distance calculator (formula 2) (47), respectively. A ≥96% ANI (31) or ≥70.0% isDDH (31, 47) was used as the cutoff to define a bacterial species. AAI was calculated between each pair of genome sequences using CompareM v0.1.2 (56) with the recommended parameters.

### Data availability.

The nearly complete 16S rRNA gene sequences, partial *rpoB* sequences, and the whole-genome shotgun projects of strains *A. tianfuensis* WCHAc060012^T^ and *A. rongchengensis* WCHAc060115^T^ have been deposited at DDBJ/ENA/GenBank under accession no. MK796537, MK796539, MK805088, MK805090, RAXV00000000, and RAXT00000000 (Table 2).

## References

[1] Brisou J, Prevot AR. 1954. Studies on bacterial taxonomy. X. The revision of species under *Acromobacter* group. Ann Inst Pasteur 86:722–728. 13197842

[2] Baumann P. 1968. Isolation of *Acinetobacter* from soil and water. J Bacteriol 96:39–42. doi:10.1128/JB.96.1.39-42.1968. 4874313 PMC252249

[3] Jung J, Park W. 2015. *Acinetobacter* species as model microorganisms in environmental microbiology: current state and perspectives. Appl Microbiol Biotechnol 99:2533–2548. doi:10.1007/s00253-015-6439-y. 25693672

[4] de Berardinis V, Durot M, Weissenbach J, Salanoubat M. 2009. *Acinetobacter baylyi* ADP1 as a model for metabolic system biology. Curr Opin Microbiol 12:568–576. doi:10.1016/j.mib.2009.07.005. 19709925

[5] Nemec A, Musílek M, Maixnerová M, De Baere T, van der Reijden TJ, Vaneechoutte M, Dijkshoorn L. 2009. *Acinetobacter beijerinckii* sp. nov. and *Acinetobacter gyllenbergii* sp. nov., haemolytic organisms isolated from humans. Int J Syst Evol Microbiol 59:118–124. doi:10.1099/ijs.0.001230-0. 19126734

[6] Nemec A, Dijkshoorn L, Cleenwerck I, De Baere T, Janssens D, Van Der Reijden TJ, Jezek P, Vaneechoutte M. 2003. *Acinetobacter parvus* sp. nov., a small-colony-forming species isolated from human clinical specimens. Int J Syst Evol Microbiol 53:1563–1567. doi:10.1099/ijs.0.02631-0. 13130049

[7] Bernards AT, de Beaufort AJ, Dijkshoorn L, Van Boven CPA. 1997. Outbreak of septicaemia in neonates caused by *Acinetobacter junii* investigated by amplified ribosomal DNA restriction analysis (ARDRA) and four typing methods. J Hosp Infect 35:129–140. doi:10.1016/S0195-6701(97)90101-8. 9049817

[8] Nemec A, Dijkshoorn L, JežEk P. 2000. Recognition of two novel phenons of the genus *Acinetobacter* among non-glucose-acidifying isolates from human specimens. J Clin Microbiol 38:3937–3941. doi:10.1128/JCM.38.11.3937-3941.2000. 11060048 PMC87521

[9] Zong Z. 2020. Genome-based taxonomy for bacteria: a recent advance. Trends Microbiol 28:871–874. doi:10.1016/j.tim.2020.09.007. 32980201

[10] Parks DH, Chuvochina M, Chaumeil PA, Rinke C, Mussig AJ, Hugenholtz P. 2020. A complete domain-to-species taxonomy for Bacteria and Archaea. Nat Biotechnol 38:1079–1086. doi:10.1038/s41587-020-0501-8. 32341564

[11] Parte AC. 2018. LPSN—list of prokaryotic names with standing in nomenclature (bacterio.net), 20 years on. Int J Syst Evol Microbiol 68:1825–1829. doi:10.1099/ijsem.0.002786. 29724269

[12] Parker CT, Tindall TJ, Garrity GM. 2019. International Code of Nomenclature of Prokaryotes. Prokaryotic code (2008 revision). Int J Syst Evol Microbiol 69:S1–S111. doi:10.1099/ijsem.0.000778. 26596770

[13] Han R-H, Lee J-E, Yoon S-H, Kim G-B. 2020. *Acinetobacter pullicarnis* sp. nov. isolated from chicken meat. Arch Microbiol 202:727–732. doi:10.1007/s00203-019-01785-y. 31792599

[14] Elnar A, Kim M, Lee J, Han R, Yoon S, Lee G, Yang S, Kim G. 2020. *Acinetobacter pullorum* sp. nov., isolated from chicken meat. J Microbiol Biotechnol 30:526–532. doi:10.4014/jmb.2002.02033. 32238766 PMC9728200

[15] Carvalheira A, Gonzales-Siles L, Salvà-Serra F, Lindgren Å, Svensson-Stadler L, Thorell K, Piñeiro-Iglesias B, Karlsson R, Silva J, Teixeira P, Moore E. 2020. *Acinetobacter portensis* sp. nov. and *Acinetobacter guerrae* sp. nov., isolated from raw meat. Int J Syst Evol Microbiol 70:4544–4554. doi:10.1099/ijsem.0.004311. 32618559

[16] Qin J, Feng Y, Lu X, Zong Z. 2020. Characterization of *Acinetobacter chengduensis* sp. nov., isolated from hospital sewage and capable of acquisition of carbapenem resistance genes. Syst Appl Microbiol 43:126092. doi:10.1016/j.syapm.2020.126092. 32690195

[17] Zhu W, Dong K, Yang J, Lu S, Lai XH, Pu J, Jin D, Huang Y, Zhang S, Zhou J, Huang Y, Xu J. 2020. *Acinetobacter lanii* sp. nov., *Acinetobacter shaoyimingii* sp. nov. and *Acinetobacter wanghuae* sp. nov., isolated from faeces of Equus kiang. Int J Syst Evol Microbiol 71. doi:10.1099/ijsem.0.004567. 33196408

[18] Acer Ö, Güven K, Poli A, Di Donato P, Leone L, Buono L, Güven RG, Nicolaus B, Finore I. 2020. *Acinetobacter mesopotamicus* sp. nov., petroleum-degrading bacterium, isolated from petroleum-contaminated soil in Diyarbakir, in the southeast of Turkey. Curr Microbiol 77:3192–3200. doi:10.1007/s00284-020-02134-9. 32725341

[19] Nemec A. 2021. Strain “*Acinetobacter mesopotamicus*” GC2 does not represent a novel species, but belongs to the species *Acinetobacter lwoffii* as revealed by whole-genome sequence-based analysis. Curr Microbiol 78:369–370. doi:10.1007/s00284-020-02275-x. 33136203

[20] Nemec A, Radolfova-Krizova L. 2017. *Acinetobacter guangdongensis* Feng et al. 2014 is a junior heterotypic synonym of *Acinetobacter indicus* Malhotra et al. 2012. Int J Syst Evol Microbiol 67:4080–4082. doi:10.1099/ijsem.0.002251. 28884669

[21] Vaneechoutte M, De Baere T, Nemec A, Musilek M, van der Reijden TJ, Dijkshoorn L. 2008. Reclassification of *Acinetobacter grimontii* Carr et al. 2003 as a later synonym of *Acinetobacter junii* Bouvet and Grimont 1986. Int J Syst Evol Microbiol 58:937–940. doi:10.1099/ijs.0.65129-0. 18398198

[22] Dunlap CA, Rooney AP. 2018. *Acinetobacter dijkshoorniae* is a later heterotypic synonym of *Acinetobacter lactucae*. Int J Syst Evol Microbiol 68:131–132. doi:10.1099/ijsem.0.002470. 29106350

[23] Wu W, Feng Y, Zong Z. 2020. Precise species identification for *Enterobacter*: a genome sequence-based study with reporting of two novel species, *Enterobacter quasiroggenkampii* sp. nov. and *Enterobacter quasimori* sp. nov. mSystems 5:e00527-20. doi:10.1128/mSystems.00527-20. 32753511 PMC7406230

[24] Mateo-Estrada V, Graña-Miraglia L, López-Leal G, Castillo-Ramírez S. 2019. Phylogenomics reveals clear cases of misclassification and genus-wide phylogenetic markers for *Acinetobacter*. Genome Biol Evol 11:2531–2541. doi:10.1093/gbe/evz178. 31406982 PMC6740150

[25] Lane D. 1991. 16S/23S rRNA sequencing, p 115–175. *In* Stackebrandt E, Goodfellow M (eds), Nucleic acid techniques in bacterial systematics. John Wiley and Sons, Chichester, UK.

[26] Hu Y, Feng Y, Zhang X, Zong Z. 2017. *Acinetobacter defluvii* sp. nov., recovered from hospital sewage. Int J Syst Evol Microbiol 67:1709–1713. doi:10.1099/ijsem.0.001847. 28211316

[27] Lee I, Chalita M, Ha S-M, Na SI, Yoon S-H, Chun J. 2017. ContEst16S: an algorithm that identifies contaminated prokaryotic genomes using 16S RNA gene sequences. Int J Syst Evol Microbiol 67:2053–2057. doi:10.1099/ijsem.0.001872. 28639931

[28] Chun J, Oren A, Ventosa A, Christensen H, Arahal DR, da Costa MS, Rooney AP, Yi H, Xu X-W, De Meyer S, Trujillo ME. 2018. Proposed minimal standards for the use of genome data for the taxonomy of prokaryotes. Int J Syst Evol Microbiol 68:461–466. doi:10.1099/ijsem.0.002516. 29292687

[29] Konstantinidis KT, Tiedje JM. 2005. Genomic insights that advance the species definition for prokaryotes. Proc Natl Acad Sci U S A 102:2567–2572. doi:10.1073/pnas.0409727102. 15701695 PMC549018

[30] Stackebrandt E, Goebel BM. 1994. Taxonomic note: a place for DNA-DNA reassociation and 16S rRNA sequence analysis in the present species definition in bacteriology. Int J Syst Evol Microbiol 44:846–849. doi:10.1099/00207713-44-4-846.

[31] Richter M, Rosselló-Móra R. 2009. Shifting the genomic gold standard for the prokaryotic species definition. Proc Natl Acad Sci U S A 106:19126–19131. doi:10.1073/pnas.0906412106. 19855009 PMC2776425

[32] Krizova L, Maixnerova M, Šedo O, Nemec A. 2015. *Acinetobacter albensis* sp. nov., isolated from natural soil and water ecosystems. Int J Syst Evol Microbiol 65:3905–3912. doi:10.1099/ijsem.0.000511. 26245775

[33] Nemec A, Radolfova-Krizova L, Maixnerova M, Sedo O. 2017. *Acinetobacter colistiniresistens* sp. nov. (formerly genomic species 13 *sensu* Bouvet and Jeanjean and genomic species 14 *sensu* Tjernberg and Ursing), isolated from human infections and characterized by intrinsic resistance to polymyxins. Int J Syst Evol Microbiol 67:2134–2141. doi:10.1099/ijsem.0.001903. 28671519

[34] Barco RA, Garrity GM, Scott JJ, Amend JP, Nealson KH, Emerson D. 2020. A genus definition for bacteria and archaea based on a standard genome relatedness index. mBio 11:e02475-19. doi:10.1128/mBio.02475-19. 31937639 PMC6960282

[35] Konstantinidis KT, Tiedje JM. 2007. Prokaryotic taxonomy and phylogeny in the genomic era: advancements and challenges ahead. Curr Opin Microbiol 10:504–509. doi:10.1016/j.mib.2007.08.006. 17923431

[36] Carvalheira A, Silva J, Teixeira P. 2021. *Acinetobacter* spp. in food and drinking water—a review. Food Microbiol 95:103675. doi:10.1016/j.fm.2020.103675. 33397609

[37] Al Atrouni A, Joly-Guillou ML, Hamze M, Kempf M. 2016. Reservoirs of non-*baumannii Acinetobacter* species. Front Microbiol 7:49. doi:10.3389/fmicb.2016.00049. 26870013 PMC4740782

[38] Wong D, Nielsen TB, Bonomo RA, Pantapalangkoor P, Luna B, Spellberg B. 2017. Clinical and pathophysiological overview of *Acinetobacter* infections: a century of challenges. Clin Microbiol Rev 30:409–447. doi:10.1128/CMR.00058-16. 27974412 PMC5217799

[39] Murray AE, Freudenstein J, Gribaldo S, Hatzenpichler R, Hugenholtz P, Kämpfer P, Konstantinidis KT, Lane CE, Papke RT, Parks DH, Rossello-Mora R, Stott MB, Sutcliffe IC, Thrash JC, Venter SN, Whitman WB, Acinas SG, Amann RI, Anantharaman K, Armengaud J, Baker BJ, Barco RA, Bode HB, Boyd ES, Brady CL, Carini P, Chain PSG, Colman DR, DeAngelis KM, de los Rios MA, Estrada-de los Santos P, Dunlap CA, Eisen JA, Emerson D, Ettema TJG, Eveillard D, Girguis PR, Hentschel U, Hollibaugh JT, Hug LA, Inskeep WP, Ivanova EP, Klenk H-P, Li W-J, Lloyd KG, Löffler FE, Makhalanyane TP, Moser DP, Nunoura T, Palmer M, et al. 2020. Roadmap for naming uncultivated Archaea and Bacteria. Nat Microbiol 5:987–994. doi:10.1038/s41564-020-0733-x. 32514073 PMC7381421

[40] Sanford RA, Lloyd KG, Konstantinidis KT, Löffler FE. 2021. Microbial taxonomy run amok. Trends Microbiol 29:394–404. doi:10.1016/j.tim.2020.12.010. 33546975

[41] Katoh K, Misawa K, Kuma K, Miyata T. 2002. MAFFT: a novel method for rapid multiple sequence alignment based on fast Fourier transform. Nucleic Acids Res 30:3059–3066. doi:10.1093/nar/gkf436. 12136088 PMC135756

[42] Felsenstein J. 1981. Evolutionary trees from DNA sequences: a maximum likelihood approach. J Mol Evol 17:368–376. doi:10.1007/BF01734359. 7288891

[43] Stamatakis A. 2014. RAxML version 8: a tool for phylogenetic analysis and post-analysis of large phylogenies. Bioinformatics 30:1312–1313. doi:10.1093/bioinformatics/btu033. 24451623 PMC3998144

[44] Bankevich A, Nurk S, Antipov D, Gurevich AA, Dvorkin M, Kulikov AS, Lesin VM, Nikolenko SI, Pham S, Prjibelski AD, Pyshkin AV, Sirotkin AV, Vyahhi N, Tesler G, Alekseyev MA, Pevzner PA. 2012. SPAdes: a new genome assembly algorithm and its applications to single-cell sequencing. J Comput Biol 19:455–477. doi:10.1089/cmb.2012.0021. 22506599 PMC3342519

[45] Parks D, Imelfort M, Skennerton C, Hugenholtz P, Tyson G. 2015. CheckM: assessing the quality of microbial genomes recovered from isolates, single cells, and metagenomes. Genome Res 25:1043–1055. doi:10.1101/gr.186072.114. 25977477 PMC4484387

[46] Jain C, Rodriguez RL, Phillippy AM, Konstantinidis KT, Aluru S. 2018. High throughput ANI analysis of 90K prokaryotic genomes reveals clear species boundaries. Nat Commun 9:5114. doi:10.1038/s41467-018-07641-9. 30504855 PMC6269478

[47] Meier-Kolthoff JP, Auch AF, Klenk HP, Goker M. 2013. Genome sequence-based species delimitation with confidence intervals and improved distance functions. BMC Bioinformatics 14:60. doi:10.1186/1471-2105-14-60. 23432962 PMC3665452

[48] Ankenbrand MJ, Keller A. 2016. bcgTree: automatized phylogenetic tree building from bacterial core genomes. Genome 59:783–791. doi:10.1139/gen-2015-0175. 27603265

[49] Seemann T. 2014. Prokka: rapid prokaryotic genome annotation. Bioinformatics 30:2068–2069. doi:10.1093/bioinformatics/btu153. 24642063

[50] Hyatt D, Chen GL, Locascio PF, Land ML, Larimer FW, Hauser LJ. 2010. Prodigal: prokaryotic gene recognition and translation initiation site identification. BMC Bioinformatics 11:119. doi:10.1186/1471-2105-11-119. 20211023 PMC2848648

[51] Bayliss SC, Thorpe HA, Coyle NM, Sheppard SK, Feil EJ. 2019. PIRATE: a fast and scalable pangenomics toolbox for clustering diverged orthologues in bacteria. Gigascience 8:giz119. doi:10.1093/gigascience/giz119. 31598686 PMC6785682

[52] Borowiec ML. 2016. AMAS: a fast tool for alignment manipulation and computing of summary statistics. PeerJ 4:e1660. doi:10.7717/peerj.1660. 26835189 PMC4734057

[53] Radolfova-Krizova L, Maixnerova M, Nemec A. 2016. *Acinetobacter pragensis* sp. nov., found in soil and water ecosystems. Int J Syst Evol Microbiol 66:3897–3903. doi:10.1099/ijsem.0.001285. 27392433

[54] Cruze JA, Singer JT, Finnerty WR. 1979. Conditions for quantitative transformation in *Acinetobacter calcoaceticus*. Curr Microbiol 3:129–132. doi:10.1007/BF02601853.

[55] Mikheenko A, Prjibelski A, Saveliev V, Antipov D, Gurevich A. 2018. Versatile genome assembly evaluation with QUAST-LG. Bioinformatics 34:i142–i150. doi:10.1093/bioinformatics/bty266. 29949969 PMC6022658

[56] Benjamin B, Chao X, Daniel HH. 2015. Fast and sensitive protein alignment using DIAMOND. Nat Methods 12:59–60. doi:10.1038/nmeth.3176. 25402007

[57] Nemec A, Radolfova-Krizova L. 2016. *Acinetobacter pakistanensis* Abbas et al. 2014 is a later heterotypic synonym of *Acinetobacter bohemicus* Krizova et al. 2014. Int J Syst Evol Microbiol 66:5614–5617. doi:10.1099/ijsem.0.001530. 27692032

