# Supplementary figures and images for: Precise Species Identification for Acinetobacter: a Genome-Based Study with Description of Two Novel Acinetobacter Species
Source: mSystems. 2021 May 26;6(3):e00237-21. doi: 10.1128/mSystems.00237-21 (PMC8269215; doi:10.1128/mSystems.00237-21)

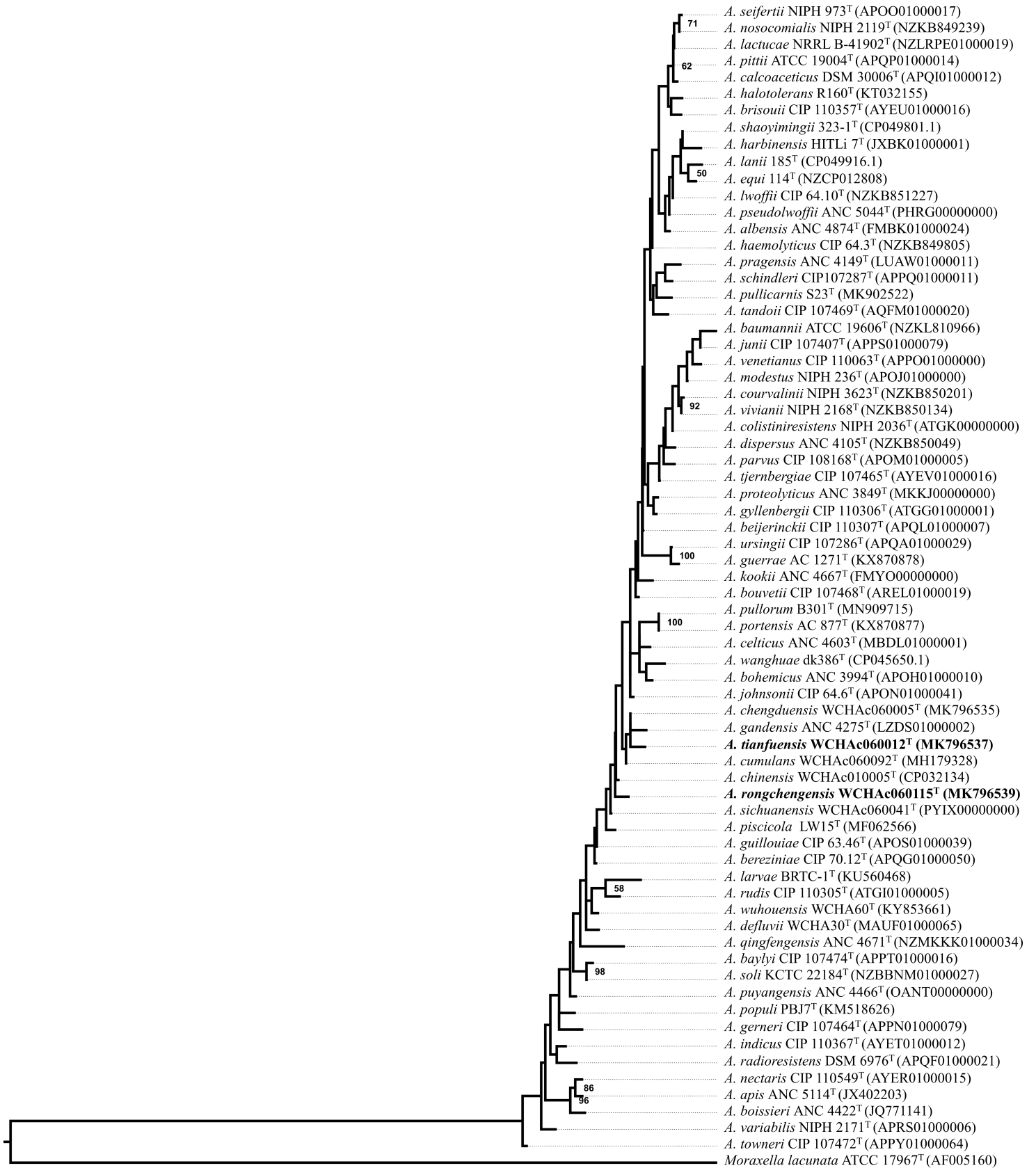

0.2

Supplement: FIG S1 [file msystems.00237-21-sf001.pdf]

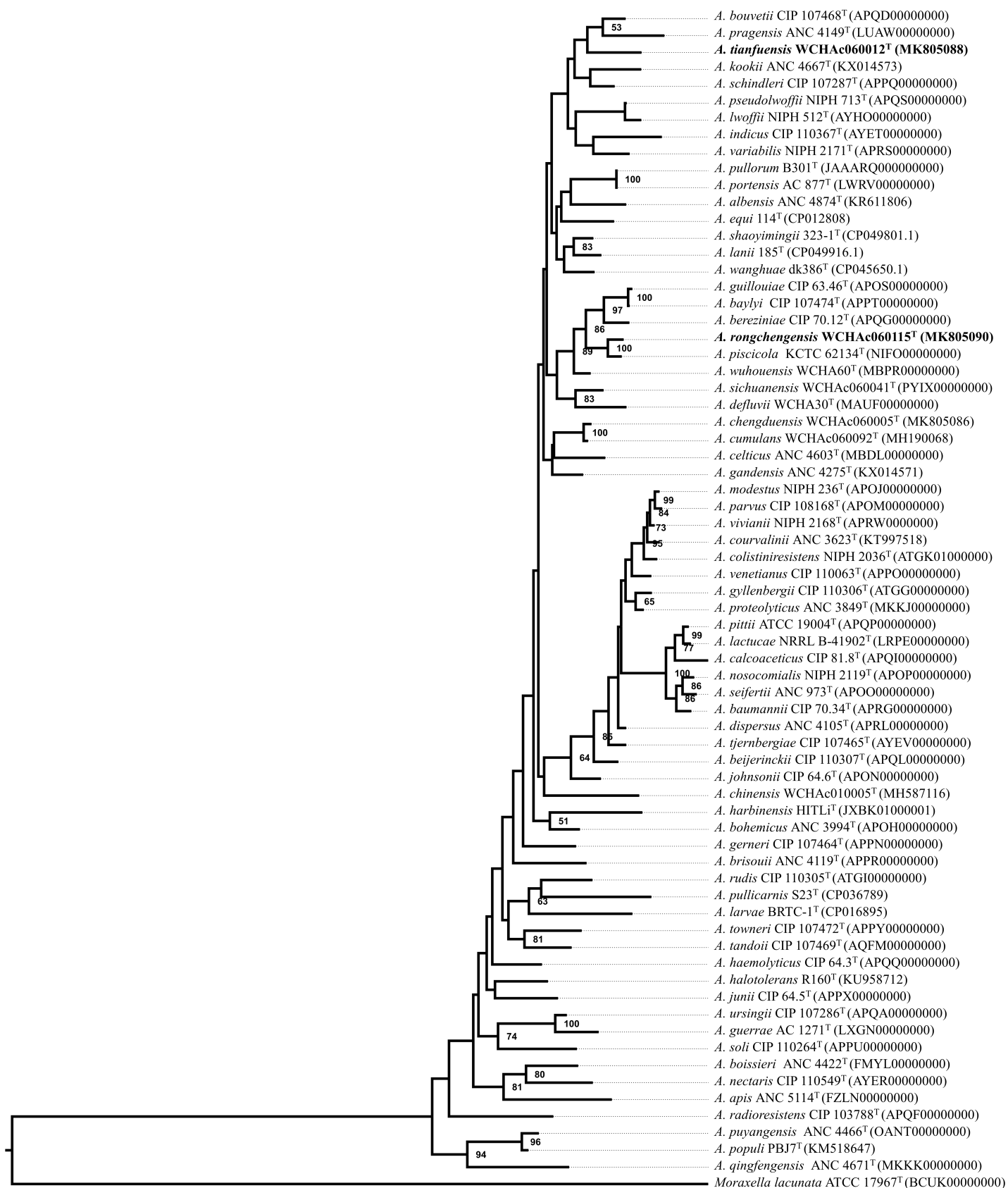

0.2

Supplement: FIG S2 [file msystems.00237-21-sf002.pdf]
