# Supplementary material for: Precise Species Identification for Acinetobacter: a Genome-Based Study with Description of Two Novel Acinetobacter Species
Source: mSystems. 2021 May 26;6(3):e00237-21. doi: 10.1128/mSystems.00237-21 (PMC8269215; doi:10.1128/mSystems.00237-21)
Supplement: TABLE S1 [file msystems.00237-21-st001.pdf]

Table S1. Antimicrobial resistance genes of *A. tianfuensis* WCHAc060012^T and *A. rongchengensis* WCHAc060115^T.

	Genes mediating resistance to				
	β-lactams	Aminoglycosides	Sulphonamides	Tetracyclines	Macrolides
WCHAc060012^T	-	*aph(3'')-Ib*, *aph(6)-Id*	*sul2*	*tet(39)*	*mph(E)*, *msr(E)*
WCHAc060115^T	bla_{NDM-1}, bla_{OXA-58}	*aph(3')-VIa*	*sul2*	-	*mph(E)*, *msr(E)*

-, not found
