# Supplementary material for: Precise Species Identification for Acinetobacter: a Genome-Based Study with Description of Two Novel Acinetobacter Species
Source: mSystems. 2021 May 26;6(3):e00237-21. doi: 10.1128/mSystems.00237-21 (PMC8269215; doi:10.1128/mSystems.00237-21)
Supplement: TABLE S2 [file msystems.00237-21-st002.pdf]

Table S2. The characteristics of strains of *A. pullorum* and *A. portensis*.

Characteristics	*A. pullorum*	*A. portensis*			
Strains	B301^{T}	AC 877^{T}	AC 1335	AC 1301	AC 1123
Catalase	+	+	+	+	+
Oxidase	-	-	-	-	-
Motility	-	-	-	-	-
Growth at 37 °C	-	-	-	-	-
Growth at 35 °C	+	ND	ND	ND	ND
Growth at 32 °C	+	ND	ND	ND	ND
Growth at 30 °C	+	+	+	+	+
Growth at 20 °C	+	+	+	+	+
Growth on R2A, 30 °C	+	+	+	+	+
Growth on TSA, 30 °C	+	+	+	+	+
0.5 % NaCl	+	+	+	+	+
1.5 % NaCl	+	+	+	+	+
3.0 % NaCl	-	+	+	+	+
6.0 % NaCl	-	-	-	-	-
Acidification of D-glucose	+	-	-	-	-
Hemolysis of horse blood	ND	-	-	-	-
Liquefaction of gelatin	-	-	-	-	-
Assimilation of					
Acetate	ND	+	+	+	+
trans-Aconitate	ND	-	-	-	-
Adipate	ND	-	-	-	-
β-Alanine	+	-	-	-	-
4-Aminobutyrate	ND	-	-	-	-
L-Arabinose	ND	-	-	-	-
L-Arginine	ND	-	-	-	-
L-Aspartate	ND	+	+	+	+
Benzoate	ND	-	-	-	-
2,3-Butanediol	ND	-	-	-	-
Citrate (Simmons')	+	+	+	+	+
Ethanol	ND	+	+	+	-
Gentisate	ND	-	+	-	-
D-Gluconate	ND	-	-	-	-
D-Glucose	+	-	-	-	-
L-Glutamate	ND	+	+	+	+
Histamine	ND	-	-	-	-
L-Histidine	+	+	+	+	+
4-Hydroxybenzoate	-	-	-	-	-
DL-Lactate	ND	+	+	+	+
L-Leucine	ND	-	-	-	-
D-Malate	+	ND	ND	ND	ND

Malonate	-	-	-	-	-
L-Ornithine	ND	-	-	-	-
L-Phenylalanine	ND	-	-	-	-
Putrescine	ND	-	-	-	-
D-Ribose	ND	-	-	-	-
L-Tartrate	ND	-	-	-	-
Tryptamine	ND	-	-	-	-
Genome					
Accession no.	JAAARQ000000000	LWRV00000000	NA	NA	NA
Completeness (%)†	98.86	98.8	NA	NA	NA
Contamination (%)†	1.35	0.89	NA	NA	NA
Strain heterogeneity (%)†	14.29	0	NA	NA	NA
G+C content (%)§	37.0	36.6	NA	NA	NA
Contigs	3	123	NA	NA	NA
Largest contig (bp)§	3,059,385	141,290	NA	NA	NA
Total length§	3,102,684	2,897,281	NA	NA	NA
N*50*§	3,059,385	61,636	NA	NA	NA
L*50*§	1	16	NA	NA	NA
Genome coverage	316	206	NA	NA	NA
Protein-coding genes	2,835	2,649	NA	NA	NA
rRNA genes	21	3	NA	NA	NA
tRNA genes	80	64	NA	NA	NA

Characteristics are scored as: +, positive reaction; −, negative reaction, ND, not determined and NA, not available.

†These were determined using CheckM (1).

§The G+C contents, largest contig, total length, N*50* and L*50* were calculated using Quast (2).

Data for species are from references (3, 4).

References

1. Parks D, Imelfort M, Skennerton C, Hugenholtz P, Tyson G. 2015. CheckM: assessing the quality of microbial genomes recovered from isolates, single cells, and metagenomes. Genome Research 25:1043–1055.

2. Mikheenko A, Prjibelski A, Saveliev V, Antipov D, Gurevich A. 2018. Versatile genome assembly evaluation with QUAST-LG. Bioinformatics 34:i142-i150.

3. Carvalheira A, Gonzales-Siles L, Salvà-Serra F, Lindgren Å, Svensson-Stadler L, Thorell K, Piñeiro-Iglesias B, Karlsson R, Silva J, Teixeira P, Moore E. 2020. *Acinetobacter portensis* sp. nov. and Acinetobacter guerrae sp. nov., isolated from raw meat. Int J Syst Evol Microbiol doi:doi: 10.1099/ijsem.0.004311.:doi: 10.1099/ijsem.0.004311.

4. Elnar A, Kim M, Lee J, Han R, Yoon S, Lee G, Yang S, Kim G. 2020. *Acinetobacter pullorum* sp. nov., isolated from chicken meat. J Microbiol Biotechnol 30:526-532.
