# Supplementary material for: Precise Species Identification for Acinetobacter: a Genome-Based Study with Description of Two Novel Acinetobacter Species
Source: mSystems. 2021 May 26;6(3):e00237-21. doi: 10.1128/mSystems.00237-21 (PMC8269215; doi:10.1128/mSystems.00237-21)
Supplement: TABLE S3 [file msystems.00237-21-st003.pdf]

Table S3. The 55 *Acinetobacter* genomes with misidentified species in NCBI

**Strain**	**Accession number**	**NCBI species**	**Precise species**	**ANI, %**
TG31977	GCA_003978795.1	*A. baumannii*	*A. courvalinii*	99.86
4300STDY7045758	GCA_900495365.1	*A. baumannii*	*A. nosocomialis*	98.05
4300STDY7045696	GCA_900494785.1	*A. baumannii*	*A. nosocomialis*	98.04
4300STDY7045796	GCA_900495745.1	*A. baumannii*	*A. nosocomialis*	97.98
4300STDY7045717	GCA_900494855.1	*A. baumannii*	*A. nosocomialis*	97.8
4300STDY7045734	GCA_900495105.1	*A. baumannii*	*A. nosocomialis*	97.79
4300STDY7045890	GCA_900496715.1	*A. baumannii*	*A. nosocomialis*	97.78
4300STDY7045889	GCA_900496685.1	*A. baumannii*	*A. nosocomialis*	97.67
4300STDY7045887	GCA_900496515.1	*A. baumannii*	*A. pittii*	97.05
4300STDY7045719	GCA_900495015.1	*A. baumannii*	*A. pittii*	96.98
1	GCA_000805365.1	*A. baumannii*	*A. seifertii*	96.89
DE0058	GCA_008764415.1	*A. baumannii*	*A. courvalinii*	96.4
WPB103	GCA_003945465.1	*A. baumannii*	Taxon 45	96.8
SFB21	GCA_902753875.1	*A. bouvetii*	Taxon 90	100
ANC 3811	GCA_000399665.1	*A. calcoaceticus*	Taxon 49	100
JUb89	GCA_004345325.1	*A. calcoaceticus*	Taxon 81	100
GK2	GCA_001510805.1	*A. calcoaceticus*	Taxon 53	100
A. calcoaceticus str. 2117	GCA_900520355.1	*A. calcoaceticus*	Taxon 53	96.34
NIPH 1859	GCA_000369765.1	*A. colistiniresistens*	Taxon 44	100
NCCP 16014	GCA_009884975.1	*A. dispersus*	Taxon 43	96.83
NCCP 16015	GCA_009884295.1	*A. gyllenbergii*	*A. proteolyticus*	97.89
KCRI-45	GCA_900406805.1	*A. haemolyticus*	Taxon 44	96.51
KCRI-348C	GCA_900406815.1	*A. haemolyticus*	Taxon 68	100
MII	GCA_000761495.1	*A. idrijaensis*	*A. lwoffii*	96.08
AJ-1	GCA_013186005.1	*A. johnsonii*	Taxon 71	98.51
AJ1	GCA_012371395.1	*A. johnsonii*	Taxon 71	98.6
ANC 3681	GCA_000368805.1	*A. johnsonii*	Taxon 42	100
MB44	GCA_001483265.1	*A. johnsonii*	Taxon 52	100
XBB1	GCA_001484935.1	*A. johnsonii*	Taxon 52	96.03
LXL_C1	GCA_003335165.1	*A. johnsonii*	Taxon 52	99.59
AJ_082	GCA_003939325.1	*A. johnsonii*	Taxon 74	100
AJ_385	GCA_003940285.1	*A. johnsonii*	Taxon 52	96.1
IC001	GCA_003952785.1	*A. johnsonii*	Taxon 75	100
M19	GCA_004337595.1	*A. johnsonii*	Taxon 52	96.39
JH7	GCA_009823385.1	*A. johnsonii*	Taxon 54	96.11
18QD2AZ57W	GCA_009828985.1	*A. johnsonii*	Taxon 54	96.2
B23	GCA_012271875.1	*A. johnsonii*	Taxon 54	96.03
C6	GCA_900162715.1	*A. johnsonii*	Taxon 42	96
AJ01M	GCA_900199565.1	*A. johnsonii*	Taxon 52	96.03
TUM15403	GCA_009003975.1	*A. oleivorans*	Taxon 46	99.77
TUM15548	GCA_009013815.1	*A. oleivorans*	Taxon 46	96.81
CM11	GCA_000962795.1	*A. parvus*	*A. modestus*	96.89
C1T1-2_a	GCA_004152775.1	*A. piscicola*	Taxon 76	100
AS012629	GCA_010612445.1	*A. pittii*	*A. lactucae*	97.69
ANC 4050	GCA_000399685.1	*A. pittii*	Taxon 48	100
LC510	GCA_001005885.2	*A. pittii*	Taxon 66	97.05
ABBL016	GCA_001432505.1	*A. pittii*	Taxon 51	100
PR366	GCA_002137095.1	*A. pittii*	Taxon 62	100
UBA4954	GCA_002397425.1	*A. pittii*	Taxon 62	97.52
KCJK7889	GCA_003053325.1	*A. pittii*	Taxon 66	100
2012N08-034	GCA_003359215.2	*A. pittii*	Taxon 72	100
SE63	GCA_006965565.1	*A. tandoii*	Taxon 65	96.14
SC36	GCA_002795165.1	*A. tandoii*	Taxon 65	100
5-2Ac02	GCA_001758345.1	*A. towneri*	Taxon 47	97.2
SCsl29	GCA_009822135.1	*A. variabilis*	Taxon 86	100
